# A survey to understand farmers' perceptions and risk factors for hoof diseases including footrot in sheep in New South Wales, Australia

**DOI:** 10.3389/fvets.2022.1000295

**Published:** 2022-10-21

**Authors:** Karen Smith, Richard J. Whittington, Alexandra C. Green, Navneet K. Dhand, Alicia Moses, Annie Grove, Tegan Thane, Om P. Dhungyel

**Affiliations:** Farm Animal Health, Faculty of Science, Sydney School of Veterinary Science, The University of Sydney, Camden, NSW, Australia

**Keywords:** footrot, sheep, hoof, risk factor, New South Wales

## Abstract

The aims of this study were to develop an understanding of farmers**'** perceptions and risk factors for footrot, including its less severe forms, and other hoof diseases in sheep in New South Wales (NSW). A questionnaire was developed and administered to sheep farmers in Local Land Services (LLS) regions across NSW. LLS staff selected sheep farmers who met the inclusion criteria which included farmers with a minimum of 100 sheep, a history of having had foot problems in their flock or having expressed an interest in improving sheep health and production. Farmers completed the questionnaire either by telephone or *via* the REDCap online survey platform. Descriptive analyses and multivariable logistic regression models were created. The survey was completed by 43 sheep farmers with a median farm size of 1,500 Ha and flock size of 2,300; footrot was present on 39% of farms while 75.6% had other hoof diseases. A flock of >3,000 sheep were more likely to have footrot than a smaller flock (OR = 11.99, 90% CI = 3.02–63.92, *P*-value = 0.005) and footrot was less likely to be present on farms when an Animal Health Statement was requested while purchasing sheep (OR = 0.10, 90% CI = 0.01–0.56, *P*-value = 0.04). Hoof conditions other than footrot were likely to be present in flocks when foot inspections were conducted at a time other than weekly inspections (OR = 0.13, 90% CI = 0.01–0.68, *P*-value = 0.04) and flocks kept on undulating ground were more likely to have diseases other than footrot compared to those kept on flat ground (OR = 3.72, 90% CI = 1.02–15.80, *P*-value = 0.09). Most farmers agreed that footrot including its less severe forms can cause production losses and negatively affect animal health and welfare. Limitations of the study were the sample size and dry environmental conditions prior to and during study period in many regions of NSW which limited the expression of footrot.

## Introduction

Lameness is a global sheep health issue that causes significant animal welfare concerns and production losses (1, 2). There are numerous non-infectious causes of lameness in sheep, however infectious diseases, which may affect multiple feet of an animal, are the most common causes of lameness (3, 4). There are several infectious foot conditions that can cause lameness in sheep, including footrot, shelly hoof, scald as well as heel and toe abscess (3, 5).

Footrot is the primary cause of lameness in sheep flocks in many countries and is recognized as the most important hoof disease in Australia (5–7). Here, two forms of footrot are recognized for regulatory purposes: benign and virulent (8). The benign form is typically limited to the interdigital skin whilst the virulent form also involves the keratin of the hoof, where the soft tissues become separated from the hard horn resulting in “underrunning” of the hoof (5, 8). The prevalence and impact of footrot is typically greater during warm and moist environmental conditions (9).

An intermediate form of footrot has also been described and it can cause underrun lesions in susceptible animals (9). Intermediate footrot is characterized by underrun lesions in up to 10% of sheep in a flock, compared to virulent footrot when 80% may develop severe lesions during ideal environmental conditions (10–12). In 2015, virulent footrot was estimated to cost the Australian sheep industry AUD $32.3 m per annum in production losses and treatments, while the estimate for benign footrot was AUD $12.10 m annually (6). No estimates have been made of the cost of intermediate footrot. Due to a successful control program, the flock-level prevalence of virulent footrot in NSW has become low (13), but the economic losses and welfare concerns associated with intermediate and benign forms of footrot, which were not the subject of control, have become more apparent (14). In this paper we refer to intermediate and benign footrot as “less severe forms” of footrot.

There can be a social stigma attached to the detection of footrot in sheep flocks in regions of Australia, including NSW (15). Virulent footrot is a notifiable disease in NSW and a diagnosis requires the farmer to implement a mandatory eradication program, which can be expensive. Restrictions on animal movements to prevent spread of the disease also may contribute to farmers being hesitant to report the disease and therefore influence how the affected flocks are managed and treated (15, 16).

The diagnosis of foot diseases can be difficult or inaccurate due to examination of too few animals, the presence of multiple lesion types and the influence of environment on disease expression (1, 4). There are varying levels of experience and expertise amongst farmers in being able to identify lame sheep and to correctly identify the type and cause of foot lesions that may be present (4). A failure to correctly identify and diagnose foot lesions can result in the application of a treatment that may be ineffective, costly and time consuming and may result in the lameness and condition persisting in the flock (16, 17). Common treatments for footrot include one or more of the following: foot bathing using a zinc sulfate solution, topical antibiotics, parenteral antibiotics, foot paring and vaccination (17–19). The efficacy and cost of footrot treatments is variable and while foot bathing and antibiotic therapies are commonly used, they are most effective when applied under ideal management and environmental circumstances (20, 21). Serogroup-specific vaccination against footrot has a curative and prophylactic effect however, approval from the Chief Veterinary Officer is required for a vaccine to be used in NSW (22, 23). There are considerable challenges in controlling and eradicating footrot, including its less severe forms (24, 25). Farm management and animal husbandry practices are critical to the prevention and control of the disease (24).

The health and welfare of sheep is dependent on several factors including their environment and animal husbandry practices (26). Farmers make decisions about farm and animal management based on the practical and economic impact of the activity as well as their personal beliefs (27). While many management decisions are profit driven, farmer knowledge, experience and access to information are increasingly being recognized as important factors in the decision-making process (27). Surveying sheep farmers about footrot has been undertaken in other sheep producing countries to gather information on their knowledge, their management practices and to identify risk factors associated with the disease (2, 28, 29).

While workshops have been conducted in Australia to improve farmer knowledge about virulent footrot including methods of control, eradication and prevention (16, 30), farmer attitudes and knowledge about the less severe forms of the disease have not been evaluated. Furthermore, understanding the economic and welfare impacts of all forms of footrot as perceived by farmers would be advantageous because they may influence their willingness to participate in control programs. The primary aim of this study was to (i) identify risk factors associated with footrot and other hoof diseases in NSW and (ii) develop an understanding of farmers' attitudes regarding the impact of footrot including its less severe forms on sheep production and welfare.

## Materials and methods

### Identification and recruitment of participants

The target population for this study was sheep farmers within a Local Land Services (LLS) region of NSW. LLS regions provide resources and governance in each region in relation to agricultural production, biosecurity and resource management. The study population was selected as follows: LLS staff identified a selection of sheep farmers in their regions who had a minimum of 100 sheep and who they believed may be interested in participating in the study. Farmers were selected based on a history of having had foot problems in their flock or for having expressed an interest in improving sheep health and production. Farmers were contacted by telephone by LLS staff, and if willing to participate, were then contacted by researchers by telephone or email to confirm their agreement to participate. Participants were sent a $50 gift voucher to partially compensate for their time. The study was approved by the Human Research Ethics Committee at The University of Sydney (approval number 2018/218).

### Questionnaire development

The questionnaire was developed to collect information relating to: (i) property description and environment; (ii) flock details and management; (iii) footrot history; (iv) biosecurity; (v) producer perception and opinion. The questionnaire contained a combination of 56 open and closed questions and a matrix of 18 questions which used a Likert scale (Supplementary Figure 1). The questionnaire was piloted by five people including farmers, a district veterinarian and a research scientist and was refined prior to being administered to survey participants. In Australia, benign footrot refers to the form of disease which is mild, virulent footrot is associated with severe underrun lesions and lameness and scald refers to interdigital lesions in the absence of *D. nodosus*. Farmers were given the choice of completing the questionnaire by telephone or using the online REDCap (31) survey format. The survey took ~25 min to complete. The questionnaires were completed between March and September 2019 (Supplementary Figure 1).

### Sample size

In order to compare the prevalence of footrot and to be able to identify and differentiate risk factors associated with a high (20%) or low (5%) prevalence of disease at the farm level, a minimum of 172 randomly selected farmers would be required to be surveyed to achieve a power of 80% for detecting a difference in proportions of 0.15 between the high and low groups at a two-sided *P*-value of 0.05 (32).

### Data entry and management

Data were collected and entered into a spreadsheet in Microsoft Excel 2016 (Microsoft Corporation, Redmond, WA, USA). Data management, statistical analyses and figures were conducted/created using Rstudio version 1.3.1056 (33), an integrated development environment for R (34–37). Figures were generated using the “ggplot2” package for R studio (38).

Explanatory variables were created from the survey responses with the variables and categories generated for each variable described in Table 1. Explanatory variables with a numerical response (average annual rainfall and number of ram sources) were analyzed using the values provided by farmers. Otherwise, binary and multi-categorical explanatory variables were created from the farmers' responses based on quantiles or biological reasons (Table 1).

**Table 1:** The list of explanatory variables generated from the farmers' response to the survey questions and the categories generated for each variable.

**Variable number**	**Explanatory variable**	**Variable type**	**Variable categories**
1	Average rainfall	Numerical	mm of rainfall
2	Topography	Categorical	Flat
			Undulating
3	Merino sheep present	Categorical	No
			Yes
4	Flock size	Categorical	< 3,000
			≥3,000
5	Self-replacing flock	Categorical	No
			Yes
6	Ram sources	Numerical	No. of sources
7	Lameness inspection	Categorical	Other
			Weekly
8	Poor hoof conformation	Categorical	< 5%
			≥5%
9	Treated when	Categorical	No treatment
			Individual
			Mob
			Treat both mob and ind.
10	Neighbor with footrot	Categorical	No
			Yes
11	Feral animals	Categorical	No
			Yes
12	Straying sheep	Categorical	No
			Yes
13	Inspect feet at purchase	Categorical	No
			Yes
14	Request animal health statement	Categorical	No
			Yes
15	Quarantine new sheep	Categorical	No
			Yes
16	Sheep proof fence	Categorical	No
			Yes

Two binary outcome variables were generated based on the responses of survey participants: (a) the presence or absence of any clinical form of footrot in the flock (yes/no); (b) the presence or absence of other hoof conditions or lesions (yes/no) including one or more of the following: scald, hoof abscess, shelly hoof.

### Descriptive analyses

Summary statistics, including the minimum, mean, median and maximum values, as well as graphical summaries in histograms and box-and-whisker plots were created for the numerical explanatory variables. For categorical explanatory variables, contingency tables were created and the percentage of responses received for each category of the variable was compared to each of the outcome variables. Explanatory variables with more than 10% of their values missing were excluded prior to any further analyses; 4 were excluded and 16 explanatory variables were retained (Table 1).

### Logistic regression analyses

A series of univariable binary logistic regression models were fitted to determine whether there was an association between the 16 explanatory variables and either of the outcome variables; the presence of any form of footrot or the presence of other hoof diseases. Explanatory variables with a Chi-square *P*-value of ≤ 0.3 were retained for further analysis, with the 90% confidence intervals calculated on the odds ratio scale for each variable. Collinearity between the explanatory variables was assessed using the Spearman's rank correlation co-efficient; variables were deemed to be collinear if they yielded a value >0.7 (Supplementary Table 1). Multivariable logistic regression models were then created for each of the outcome variables using a manual forward stepwise selection method. Explanatory variables with an association with the outcome value were retained in the final models with the *P-*value cut off set at ≥0.1 for both outcome variables. The goodness of fit of the final models for both outcome variables were assessed using the Hosmer–Lemeshow test (34).

### Categorizing farmer responses to matrix statements

Statements included in the matrix questions broadly covered two categories specifically relating to intermediate and benign footrot: (i) farmer perceptions (ii) the effect of disease on production and the availability of veterinary resources. The farmers' responses to the statements were condensed into three categories: (i) disagree (ii) agree (iii) neither agree nor disagree. The number and percentage of responses given for each of the three categories was reported.

### Farmer comments

Farmers provided comments in relation to footrot, other hoof diseases, biosecurity and the treatment and management of footrot and lameness. The most frequently used words in the additional comments provided by the farmers relating to hoof disease, treatment and management were identified.

## Results

### Descriptive analyses

**Figure 1:**
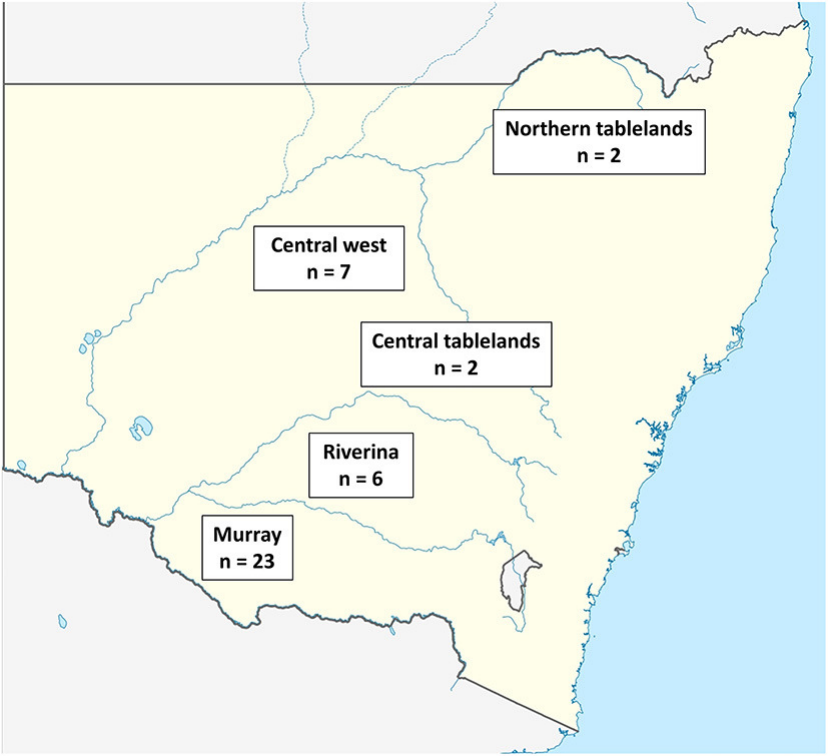
Map of NSW showing the approximate location of the LLS regions within NSW where participants completed the questionnaire and the number of participating farmers in each region.

The total number of farmers contacted by LLS staff was not provided; however, 56 farmers agreed to be contacted by researchers. This is ~2.3% (56/2,345) of the sheep farmers reported to be in NSW in 2018 (39). The questionnaire was completed by 76.8% (43/56) of the farmers who had agreed to be contacted by researchers. The Murray LLS region had the highest number of participants (23/43), followed by the Central West LLS (7/43) and Riverina LLS (6/43) while there were fewer from the Central tablelands LLS (2/43) and Northern tablelands LLS (2/43). Three participants who completed the survey did not report their LLS region (Figure 1).

**Figure 2:**
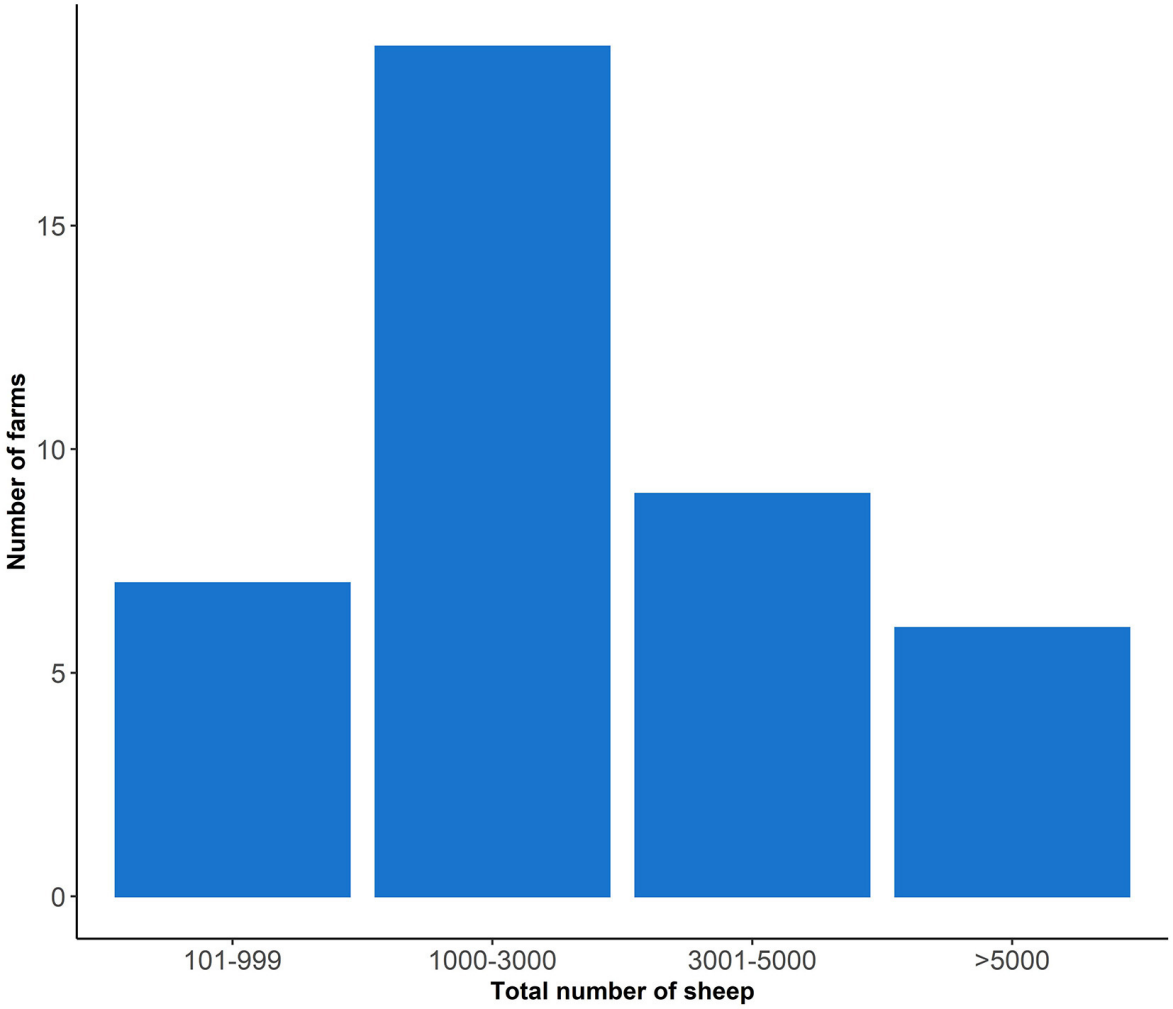
The total number of sheep owned by 41 farmers who participated in the study; two farmers did not report their flock size.

The median farm size was 1,500 hectares (Ha) and the median flock size was 2,300 sheep (Table 2). Two farmers did not report their flock size but 46.34% (19/41) had a flock size of between 1,000 and 3,000 sheep (Figure 2). Most sheep were grazed on improved pastures (79% of farms) (Table 2). Lamb production was the primary enterprise type (86.04%) followed by wool production (69.77%) with Merino sheep being the most common sheep breed (Table 2). In the previous year 16/41 (39%) farmers reported the presence of footrot on their farm in the previous year. Foot abscess (27/41, 65.9%) and scald (15/41, 36.6%) were the most frequently reported hoof conditions (Table 2).

**Table 2:** Summary of the farm information, production type and hoof diseases present in farms included in the survey.

**Variable**	**Category**	**No. responses**	**Min**	**Max**	**Mean**	**Median**	**“Yes” response (%)**
Farm size	(Ha)	41	31	7,000	2,017	1,500	–
Altitude	(m)	27	70	800	278	290	–
Rainfall	(mm pa)	41	200	708	525	550	–
Improved pasture	–	43	–	–	–	–	34 (79.07%)
Flock size	Total sheep	41	110	18,000	3,316	2,300	
Enterprise type	Wool	43	–	–	–	–	30 (69.77%)
	Lamb prod.	43	–	–	–	–	37 (86.05%)
	Mixed*	43	–	–	–	–	21 (48.84%)
Sheep breed	Merino	41	–	–	–	–	29 (70.73%)
	Cross-bred	42	–	–	–	–	24 (57.14%)
	Other breed	40	–	–	–	–	6 (15.00%)
Footrot present in last year	–	41	–	–	–	–	16 (39.02%)
Form of footrot	Virulent	41	–	–	–	–	8 (19.51%)
	Benign	41	–	–	–	–	8 (19.51%)
Other hoof condition	Shelly hoof	41	–	–	–	–	14 (34.15%)
	Scald	41	–	–	–	–	15 (36.59%)
	Abscess	41	–	–	–	–	27 (65.85%)

^*^Sheep and cattle.

Participants mostly agreed that the presence of the less severe forms of footrot had a negative impact on the welfare of sheep (90.6%, 39/43). Most farmers agreed that the presence of lameness due to footrot causes production (93%, 40/43) and economic (83.7%, 36/43) losses. Farmers reported a high level of confidence in their ability to identify sheep with footrot lesions (88.3%, 38/43) and considered footrot including its less severe forms to be an important sheep health issue (88.3%, 38/43). In relation to the impact of the less severe forms of footrot on their own farms, 39.5% (17/43) of farmers agreed that they were a minor issue on their own property and 37.2% (16/43) of farmers agreed they were a major problem on their property (Table 3).

**Table 3:** Summary of participant responses to statements about the less severe forms of footrot including their impact on animal welfare and production and the veterinary resources available to deal with these conditions.

**Statement**	**Disagree (*n* %)**	**Agree (*n* %)**	**Neither (*n* %)**	**N/A (*n* %)**
Quarantine procedures are important in preventing disease	6 (13.95%)	37 (86.05%)	–	–
Footrot is caused by bacteria	4 (9.30%)	36 (83.72%)	3 (6.98%)	–
It is important to examine the feet of sheep prior to purchase	6 (13.95%)	37 (86.05%)	–	–
I am confident in my ability to identify sheep with footrot	2 (4.65%)	38 (88.37%)	3 (6.98%)	–
I consider hoof health an important animal health issue in the NSW sheep industry	5 (11.63%)	38 (88.37%)	–	–
I consider footrot a minor problem on the property	20 (46.51%)	17 (39.53%)	5 (11.63%)	1 (2.33%)
I consider footrot a major problem on the property	24 (55.81%)	16 (37.21%)	2 (4.65%)	2 (4.65%)
Poor hoof health has a negative impact on the welfare of affected sheep	4 (9.30%)	39 (90.70%)	–	–
Footrot is difficult to eradicate	10 (23.26%)	29 (67.44%)	4 (9.30%)	–
Sheep on my property are at risk of footrot from infected neighboring animals	18 (41.86%)	21 (48.84%)	4 (9.30%)	–
Lameness causes production losses (i.e., reduced wool and carcase weight)	3 (6.98%)	40 (93.02%)	–	–
I consider footrot a significant source of economic loss to the NSW sheep industry	4 (9.30%)	36 (83.72%)	3 (6.98%)	–
There is sufficient online government resources about footrot	5 (11.63%)	24 (55.81%)	14 (32.56%)	–
I am happy with the services and information offered by Government veterinarians	3 (6.98%)	32 (74.42%)	8 (18.60%)	–
I am happy with the services and information offered by Private veterinarians	2 (4.65%)	35 (81.40%)	6 (13.95%)	–
Current methods of footrot management are cost effective	6 (13.95%)	20 (46.51%)	17 (39.53%)	–
Current methods of footrot management are time effective	13 (30.23%)	18 (41.86%)	12 (27.91%)	–

Biosecurity practices were considered to be important by farmers. Most (86%, 37/43) agreed with both of these statements: that quarantine procedures are important in preventing footrot; that the examination of the feet of sheep prior to purchase is important. Most farmers agreed that footrot is difficult to eradicate (67.4%, 29/43) and many responded that there is a risk of a flock being infected if a neighboring farm is infected (48.8%, 21/43) (Table 3). There was a general agreement that there are sufficient online (55.8%, 24/53) and field veterinary resources, including both government (74.4%, 32/43) and private (81.3%, 35/43) veterinary services.

The opinions of farmers toward the cost and time effectiveness of current management methods were broader than for other statements. Less than half of farmers agreed that current methods were cost (46.5%, 20/43) and time (41.8%, 18/43) effective (Table 3).

### Farmer comments and frequently reported words

Farmers generally reported having good biosecurity measures and fencing with one participant commenting “always keep your boundary fences 100% and always be vigilant.” Farmers commented that they reduce the risk of introducing footrot and disease by having “closed flocks,” breeding their own replacement ewes and only purchasing rams from “a trusted source.” There was a range of opinions and comments from farmers regarding the use of footrot treatments and the feasibility of being able to eradicate footrot from a flock. One participant reported that if animals on his property were to have footrot, they “would cull or sell all stock” as opposed to treating as he believes it would be extremely difficult to eradicate the disease. Another farmer's comments were in agreement with this approach saying that they use foot-bathing with zinc sulfate to treat and reduce the prevalence of scald in the flock however they would prefer complete elimination of the disease. It was stated that footrot can be eliminated from a farm if a “hard line” is taken, however the farmer acknowledged that elimination requires significant time, labor and the correct conditions for it to be successful.

Comments from nine farmers indicate that hoof inspections are typically conducted when lameness is observed within the flock. Several farmers reported that if an individual lame sheep is observed they will catch and inspect it to determine the cause of the lameness. One participant stated that they conduct hoof inspections at “crutching and over summer” and another commented that they “only inspect the feet of rams when preparing to show the animal or prior to sale.”

Words detected four or more times in the comments provided by farmers in relation to the topic footrot health and diseases were identified *via* text mining. The words which were detected in comments ≥4 times are reported in Table 4. Thirteen words were identified with the two most frequently detected words “eradication” (*n* = 9) and “scald” (*n* = 9) Whilst the next most frequently detected words were “eradication” (*n* = 7) and “cull” (*n* = 7) (Table 4).

**Table 4:** The words detected four or more times in the additional comments provided by farmers regarding footrot and other hoof diseases.

**Ranking**	**Word**	**Frequency**
1	Eradication	9
2	Scald	9
3	Vaccine	7
4	Cull	7
5	Antibiotic	6
6	Vet	6
7	Benign	5
8	Treat	5
9	Footbath	5
10	Flock	5
11	Fence	5
12	Neighbors	4
13	Wet	4

### Univariable analysis

The explanatory variables were generated from farmer responses to questions relating to (i) property description and environment; (ii) flock details and management; (iii) footrot history; (iv) biosecurity; (v) producer perception and opinion (Supplementary Figure 1). Explanatory variables were generated from questions which had a response rate of ≥90%. Based on the univariable regression models with the binary outcome of footrot (yes/no), 10 of the 16 explanatory variables had a Chi-square *P*-value of < 0.3 and were retained for further analysis (Table 5). Notably, the following factors achieved statistical significance at *P* < 0.05: Merino breed, large flock size, straying sheep, requesting an Animal Health Statement. Based on the univariable regression models with the binary outcome of the presence of absence of other hoof diseases (yes/no), eight of the 16 explanatory variables had a Chi-square *P*-value of < 0.3 and were retained for further analysis (Table 5). All explanatory variables had a Spearman's rank correlation coefficient *P*-value of < 0.7 and were therefore retained for regression analyzes.

**Table 5:** The explanatory variables and their definition/categories and the values from the univariable analyses for each outcome, footrot or other hoof diseases.

**Explanatory variable**	**Variable categories**	**Outcome variable**			
		**Presence of footrot**		**Presence of other hoof diseases**	
		**OR (90% CI)**	***P-*value**	**OR (90% CI)**	***P-*value**
Average rainfall	mm of rainfall	1.0 (0.99–1.01)	0.7	1.00 (1.00–1.01)	0.58
Topography	Flat	1.00	–	1.0	–
	Undulating	3.54 (1.16–12.01)	0.06*	3.23 (0.93–12.85)	0.12*
Merino sheep present	No	1.0	–	1.0	–
	Yes	8.93 (1. 87–93.59)	0.01*	1.12 (0.27–4.10)	0.88
Flock size	< 3,000	1.0	–	1.0	–
	≥3,000	12.83 (3.71–53.43)	< 0.01*	1.20 (0.32–4.98)	0.82
Self-replacing flock	No	1.0	–	1.0	–
	Yes	0.79 (0.24–2.78)	0.76	0.27 (0.02–1.35)	0.19*
Ram sources	No. of sources	1.43 (0.89–2.40)	0.22*	1.15 (0.68–2.05)	0.66
Lameness inspection	Other	1.0	–	1.0	–
	Weekly	2.24 (0.68–8.62)	0.27*	0.15 (0.01–0.74)	0.05*
Poor hoof conformation	< 5%	1.0	–	–	–
	≥5%	1.37 (0.44–4.43)	0.65	–	–
Treated when	No treatment	1.0	–	1.0	–
	Individual	4.12 (0.95–22.87)	0.39	2.25 (0.87–6.58)	0.19*
	Mob	3.30 (0.59–21.32)	–	2.66 (0.56–15.28)	–
	Treat both	4.12 (0.71–27.77)	–	0.29 (0.04–1.74)	–
Neighbor with footrot	No	1	–	1	–
	Yes	2.33 (0.68–8.17)	0.26*	0.3 (0.07–1.44)	0.21*
Feral animals	No	1	–	1	–
	Yes	1.20 (0.34–3.97)	0.80	1.4 (0.3–10.5)	0.70
Straying sheep	No	1.0	–	1	–
	Yes	8.70 (2.60–34.83	0.002*	2.33 (0.67–9.27)	0.26*
Inspect feet at purchase	No	1.0	–	1	–
	Yes	0.36 (0.09–1.38)	0.26*	2.78 (0.62–11.86)	0.25*
Request animal health statement	No	1.0	–	1	–
	Yes	0.10 (0.02–0.42)	0.007*	1.0 (0.18–4.17)	1.00
Quarantine new sheep	No	1.0	–	1	–
	Yes	1.64 (0.41–8.49)	0.57	2.78 (0.62–11.86)	0.25*
Sheep proof fence	No	1.0	–	1	–
	Yes	0.30 (0.08–1.07)	0.12*	0.36 (0.03–1.88)	0.34

OR, odds ratio; CI, confidence interval.

^*^Explanatory variables with a P-value < 0.3 were retained for analysis in the multivariable model.

### Multivariable analysis

There were two variables in the final multivariable logistic regression model for the outcome variable of footrot (Yes/No) and the presence of other hoof diseases (Yes/No). For both outcome variables the category “Yes” was associated with the presence of footrot or other hoof disease. The Hosmer–Lemeshow test indicated no lack of fit the final multivariable models for both outcome variables, the presence of footrot (*P* = 0.64) and the presence of other hoof diseases (*P* = 0.84).

A flock of >3,000 sheep was more likely to have footrot than a smaller flock (OR = 11.99, 90% CI = 3.02–63.92, *P*-value = 0.005) and footrot was more likely to be present on farms when the farmer did not request an Animal Health Statement when purchasing sheep (OR = 0.10, 90% CI = 0.01–0.56, *P*-value = 0.04) (Table 6). Hoof conditions other than footrot were likely to be present in flocks when foot inspections were conducted at a time other than weekly inspections (OR = 0.13, 90% CI = 0.01–0.68, *P*-value = 0.04) and flocks kept on undulating ground were more likely to have diseases other than footrot compared to those kept on flat ground (OR = 3.72, 90% CI = 1.02–15.80, *P*-value = 0.09) (Table 6).

**Table 6:** Multivariable models for the binary outcome variables: Presence of any clinical form of footrot; presence of other hoof diseases (*P*-value ≥0.1).

**Outcome**	**Variable**	**Category**	**Estimate**	**SE**	**OR**	**90% CI**		***P-*value**
						**Lower**	**Upper**	
Footrot	Flock size	Intercept	−2.58	1.53	–	–	–	0.005
		Total sheep < 3,000	0.00	–	1.00	–	–	
		Total sheep ≥ 3,000	2.48	0.90	11.99	3.02	63.92	
	Request AHS	No	0.00	–	1.00	–	–	0.04
		Yes	−2.24	1.09	0.10	0.01	0.56	
Other hoof diseases	Lameness inspection	Intercept	0.77	1.06	–	–	–	0.04
		Other	0.00	–	1.00	–	–	
		Weekly	−2.00	1.1	0.13	0.01	0.68	
	Topography	Flat	0.00	–	1.00	–	–	0.09
		Undulating	1.31	0.8	3.72	1.02	15.80	

SE, standard error; OR, odds ratio; CI, confidence interval; AHS, animal health statement.

## Discussion

There is pressure on farmers to increase production efficiency whilst maintaining high levels of animal health and welfare (40). In this context the adverse economic and welfare impacts of lameness in sheep due to infectious and non-infectious diseases has been recognized previously (1, 41) and is reflected in the responses provided by farmers in this survey. Most farmers agreed that lameness causes productivity losses, economic losses and has a negative impact on the welfare of sheep. This survey followed others conducted overseas in which information was collected from farmers on lameness and sheep health topics including treatments (42), control methods (42), farmer knowledge (43), disease prevalence (2), and the identification of risk factors for disease expression and transmission (44). The recruitment of farmers from different regions of NSW who had farms with a broad range of sizes, average annual rainfall, altitude and with different enterprise types and disease histories, resulted in selection of participants who were likely to be representative of commercial sheep farmers across the eastern part of NSW where the majority of sheep are grazed, although the northern part of this region was under-represented (see below). In this study the non-random sampling of farmers was conducted to be able to recruit sufficient participants in a relatively short period of time and therefore the cross-sectional study design was the most appropriate.

The average flock size in NSW is 2,423 which compares with the median flock size of 2,300 in this study (39). A limitation of the study was the low number of sheep farmers in NSW who participated in the study. However, the information collected reflects the prevalence of the presence of disease and impact when the survey was conducted as well as providing insight into the attitudes toward hoof health present in farmers who completed the questionnaire. Farmers were selected to participate in the study if they had expressed an interest in sheep health or had a history of foot problems. Whilst the farm size and production types are representative of farms in NSW, the selection of farmers in this manner may result in selection bias. Farmers who have experienced footrot in their flocks may have been more willing to participate in the study, biasing the estimate of the prevalence of disease at the farm level. Increasing the sample size and the selection and inclusion of farmers with and without a history of hoof diseases including footrot may be used to better represent and limit selection bias.

Footrot has been identified as a priority endemic disease due to the widespread impact on production losses and animal health and welfare (6). There is a social stigma attached to footrot within the farming community in many areas of Australia, with farmers concerned their reputation will be harmed due to the presence of the disease (15). In addition, virulent footrot is a notifiable disease in NSW, which may deter some farmers from participating in a survey due to concerns about them reporting on the presence of virulent footrot on their farm (15). That the researchers were not responsible for disease regulation in NSW and have been active in dissemination of information on sheep health to farmers in the past, which may have contributed to the high response rate in this study.

Footrot is a multifactorial disease with variations in animal susceptibility, environmental conditions and the strain(s) of *D. nodosus* present all influencing the clinical severity of disease (8, 45). Topography of undulating hills was determined to be a risk factor for the presence of foot diseases including foot abscesses, scald and shelly hoof with the level of statistical significance set at *P* < 0.1. This variable would be excluded using a level of significance set at *P* < 0.05, however, flock management including housing or maintaining animals in wet conditions may influence the prevalence of hoof diseases. Wet environmental conditions have been shown to be associated with foot abscesses in the Central Tablelands of NSW and in New Zealand (46, 47). Wet environmental conditions may also predispose animals to other foot infections such as scald (46). Topography was not associated with the presence of footrot, the expression of which is moisture dependent (12).

While flock sizes and the number of sheep in NSW had declined over several years prior to the survey due to severe drought conditions (39), having a large flock size of ≥3,000 sheep was identified as a risk factor for the presence of footrot. The size of a flock was reported to be associated with lameness in some studies of sheep flocks in the UK, whilst other studies determined there was no association (29, 44, 48). A large flock size has also been identified as a risk factor for ovine Johne's disease in Australia (49). Farmers with larger flocks have less time to manage individual animals and adopt management practices aimed at the flock rather than individual animals (50). It may take longer to identify and treat affected animals in a large flock, which may influence the prevalence of lameness and the effectiveness of the treatments used (51).

Additional explanatory variables were statistically significant in the univariable analysis examining risk factors associated with footrot, but not in the final multivariable model. This may partially be attributed to the sample size used in the study (52). For example, the presence of the Merino breed on a farm was not retained in the final model even though Merino sheep have been shown to be more susceptible to footrot than British breeds and cross-bred sheep in Australia (5, 53). The influence of pasture type and coverage, stocking density and quarantine practices were not explored in the study due to the limited number of responses received, however, these factors have been associated with footrot and lameness and are recommended to be investigated in future studies (2, 12, 44). The dry environmental conditions that existed during the survey period may have reduced the range of prevalence of footrot across flocks in NSW, diminishing expression of the known effects of sheep breed on footrot prevalence. However, the methodology of testing for collinearity between explanatory variables prior to the generation of the regression models and the use of a forward selection method did lead to the retention of variables in the model which are related to farm management (flock size) and have biosecurity significance (requesting an Animal Health Statement when purchasing sheep) (52). Animal Health Statements (National Sheep Health Declaration) may be completed by farmers when selling sheep in Australia and allow health conditions and diseases which may be present, including benign and virulent footrot, to be declared prior to sale. This allows prospective buyers to obtain information about the history of the flock prior to purchasing animals and limits the risk of purchasing animals with illness or disease.

A range of climatic conditions may arise during the seasonal changes in NSW between March to September, which was the time frame when the survey was administered. For several years prior to the survey, and during the survey, a large proportion of NSW was in drought, with northern regions of the state being severely affected. The number of sheep farmers available to complete the survey in Northern NSW was limited; some farms had been required to de-stock partially or completely due to the impact of the drought and there were other management priorities. More generally, and based on knowledge of the pathogenesis, hoof conditions in flocks across regions of NSW would likely have been influenced by the prolonged dry climatic conditions, and in particular there would likely have been a reduction in the prevalence and severity of footrot compared to wet seasons (12). While this could have diminished farmer perceptions of the importance of the disease, it was not evident in the results, a factor that might reflect the success of extension programs associated with the Footrot Strategic Plan in NSW (13).

All forms of footrot were grouped together in the univariable and multivariable analyzes, however, information relating specifically to the less severe forms intermediate and benign footrot was also collected and analyzed (Table 3). In this survey farmers agreed that the less severe clinical forms of the disease were an important health issue in the sheep industry in NSW. In relation to the impact on their own farms, 39.5% (17/43) of farmers agreed that the less severe forms of footrot were a minor issue on their farm, whilst 37.2% (16/43) felt that they were a major issue on their farm. This suggests that intermediate and benign footrot are important disease conditions that should be investigated, diagnosed and managed.

Differentiating and classifying the clinical form of footrot may be challenging, especially in the early stages of infection (11). Furthermore, to avoid regulatory action, farmers may self-diagnose and treat sheep for virulent footrot to prevent the disease being detected and diagnosed on their farm by government veterinarians. This may result in a poor response to treatments, especially if an incorrect diagnosis is made or an inappropriate treatment regime applied (15). The hoof condition scald, which is caused by *Fusobacterium necrophorum*, causes lesions limited to the interdigital skin and may be mistaken for benign footrot (17, 45). The word “scald” was identified as being frequently used by farmers, suggesting interdigital lesions are considered significant and problematic by farmers, even in drought conditions. In this study, the majority of farmers indicated they were confident in their ability to identify footrot, however, the actual ability of farmers to identify and differentiate the clinical forms of footrot was not able to be explored further in this study. The ability of British sheep farmers to identify six types of foot lesions was assessed using pictorial and written descriptions of each lesion type (4). The results of that study determined veterinarians and specialists within the sheep industry were better able to accurately identify foot lesions than farmers, with footrot being the most incorrectly used lesion name and incorrectly applied to lesions of the hoof horn (4). For these reasons the results of the current study probably reflect farmer perceptions about the entire complex of footrot, virulent, intermediate and benign. The farmers' attitudes and perceptions probably were formed when footrot became clinically apparent in their flocks, regardless of the technical veterinary classification into particular virulence categories.

Failing to identify and remove lame or affected sheep from a flock can result in a higher prevalence and severity of footrot as the affected animals are a source of contamination and infection for other susceptible animals (54). While the identification and inspection of an individual lame animal is recommended and able to be implemented in some farming enterprises, this practice would be impractical to implement on most Australian farms due to large flock sizes. While it is ideal to be able to examine and treat individual sheep within 3 days of lameness occurring, it may be difficult for farmers to catch and treat sheep in large flocks and farm areas (55). In this study the there was a significant negative association in frequency of lameness inspections and the presence of hoof conditions other than footrot, which is to be expected as the presence of hoof conditions causing lameness is likely to trigger inspections.

Foot bathing a large flock can be time and labor intensive, with farmers in this study agreeing, indicating they would prefer to use the more convenient footrot treatments of antibiotics or vaccination. While hoof paring was widely considered to be a routine husbandry procedure, it has been shown to be most effective when used selectively to trim overgrown feet and to aid in the identification of hoof lesions (17, 20). When feet are trimmed excessively and made to bleed, it may cause lameness and reduce the efficacy of topical treatments (29, 56). Recent studies have demonstrated the use of parenteral and topical antibiotics are more effective in reducing the severity of footrot lesions than foot trimming (57, 58). Footrot treatments were not explored in the study due to the limited number of responses to survey questions on the topic, however it should be considered in future studies.

In NSW, serogroup-specific vaccine may be used with approval of the Chief Veterinary Officer as part of an eradication program approved by an LLS veterinarian and is not administered as part of a routine vaccination schedule or an unregulated lameness control program (59). The whole cell, multivalent vaccine Footvax^®^ is approved and recommended to be used by farmers in the UK to control footrot, with 29.2% of farmers reported to use the vaccine in 2015 (42, 60). Footvax^®^ is also available in New Zealand and has been reported to reduce the prevalence of clinical footrot in flocks with virulent footrot, but not eliminate disease (61). Farmers in this study indicated the implementation of a vaccine program in NSW, especially one which could be applied by private veterinarians, would be beneficial as it would offer a more cost and time effective control method than those currently used. However, additional research into the efficacy of vaccines against benign and intermediate footrot is required as virulent footrot has historically been the focus of research (23, 62). A recent study in NSW suggested that serogroup-specific vaccination may be used as a tool to control and eliminate intermediate forms of footrot (63).

Biosecurity practices are essential in preventing the introduction of diseases at the farm and regional level (64). Quarantine and farm biosecurity procedures can be integrated in a disease prevention strategy or within a disease control program to prevent infection or re-infection of a flock (65) and also to reduce within-farm spread, for example where sheep are managed in different mobs. In this study most farmers agreed the quarantine of newly arrived animals was important in preventing disease on the farm. Maintaining farm boundaries and fencing and having a closed flock are important aspects of biosecurity as straying sheep may introduce or re-introduce disease from neighboring flocks (17). In this study some farmers identified neighboring animals as a potential source of infection, and almost half of the participants agreed neighboring animals were a risk of introducing footrot onto their farm.

In NSW mobs or flocks of sheep were often culled as part of an approved virulent footrot control program and while some farmers in this study stated they had culled mobs, there was a mixed response regarding the topic of treating or culling animals due to the presence of footrot. Due to the time and the difficulty associated with eradiating footrot, two farmers commented they would cull stock as opposed to treating them. The culling of individual animals in Australia is recommended for sheep with chronic infections and those that do not respond to treatments (22). However, the costs associated with treating or attempting to eradicate intermediate or benign footrot may exceed the production benefits gained (66); this could be a problem especially if treatments are not effective or if repeated treatments are needed. Whilst production losses associated with these forms of footrot are less significant than those due to virulent footrot (25, 41), farmers may be willing to justify the costs to improve the health and welfare status of their animals *per se*.

A limitation of the study included the environmental conditions prior to and at the time of the survey which were unfavorable for footrot transmission. This contributed to a low response rate to questions relating to the percentage of animals on farms with hoof diseases. The small sample size of 43 respondents was another limitation of the study. The sample of respondents may therefore not be representative of the broader New South Wales Australian sheep farmer population, so caution should be taken when generalizing the findings. Since the sample size was lower than the desired number, our study may not have been able to detect statistical differences between some groups in the data. Nonetheless, we still managed to show that the flock size and the request of an AHS prior to purchase were significantly associated with the presence of footrot, and the frequency of lameness inspections and farm topography were significantly associated with the presence of other hoof diseases, suggesting that even with low power these variables do indeed have an influence on foot diseases. To enhance the response rate of future studies, we recommend collecting data in person to ensure the questionnaire is completed in entirety, as well as recruiting participants through channels such as stock agents or industry bodies in addition to Local Land Services. Further research aimed at providing insight into to the efficacy of current treatment and control methods in Australian flocks, particularly against intermediate and benign footrot for which there is relatively little information, would also be beneficial.

## Data availability statement

The raw data supporting the conclusions of this article will be made available by the authors, without undue reservation.

## Ethics statement

The studies involving human participants were reviewed and approved by Human Ethics Committee, The University of Sydney. The patients/participants provided their written informed consent to participate in this study.

## Author contributions

KS, ND, RW, and OD conceived and designed the study and wrote the paper. KS, ACG, and ND contributed in statistical analysis. KS, AM, AG, and TT conducted surveys and collated data. All authors contributed to the article and approved the submitted version.

## Conflict of interest

The authors declare that the research was conducted in the absence of any commercial or financial relationships that could be construed as a potential conflict of interest.

## Publisher's note

All claims expressed in this article are solely those of the authors and do not necessarily represent those of their affiliated organizations, or those of the publisher, the editors and the reviewers. Any product that may be evaluated in this article, or claim that may be made by its manufacturer, is not guaranteed or endorsed by the publisher.
