# Supplementary material for: A survey to understand farmers' perceptions and risk factors for hoof diseases including footrot in sheep in New South Wales, Australia
Source: Front Vet Sci. 2022 Oct 21;9:1000295. doi: 10.3389/fvets.2022.1000295 (PMC9634747; doi:10.3389/fvets.2022.1000295)
Supplement: Supplementary file 1 [file Data_Sheet_1.docx]

Supplementary Material

**Supplementary Figure 1.** The questionnaire which was completed by sheep farmers between March and September 2019.

**Property name:**___________________________________________________________________

**Name:**___________________________________________________________________________

**Phone number:**____________________________________________________________________

**Email:**___________________________________________________________________________

**Property address:** ________________________________________________________________

**I agree to be contacted for follow-up questions if required 🞎**

**Best time to be contacted: Between** _________________ **and** ____________________

Local Land Services (LLS) region __________________________________

**SECTION 1 – Property description, Environment and management**

**Property:**

| Total area (ha) |  | Topography (Please tick) | Flat | **🞎** |
| --- | --- | --- | --- | --- |
| Altitude (m) |  |  | Undulating | **🞎** |
| Nearest BOM station |  |  | Steep | **🞎** |
| Annual rainfall (mm) |  |  |  |  |
| **%** Grazed |  |  |  |  |
| Soil type |  |  |  |  |

**Pasture:**

| Pasture type | Native species | | **🞎** | | Pasture length in an average season (mm) | |
| --- | --- | --- | --- | --- | --- | --- |
|  | Forage crops | | **🞎** | | Winter |  |
|  | Introduced perennial species | | **🞎** | | Spring |  |
|  | Improved pasture | | **🞎** | |  |  |
| Other (please specify) | |  | |  |  |  |

| Total % of property pasture coverage in an average season |  |
| --- | --- |

**Enterprise type:**

| Lamb production | **🞎** | Wool type | Superfine/Ultrafine (≤ 18.5 microns) | **🞎** |
| --- | --- | --- | --- | --- |
| Mixed (sheep and cattle) | **🞎** |  | Fine (18.6-19.5 microns) | **🞎** |
| Wool | **🞎** |  | Fine-medium (19.6-20.5 microns) | **🞎** |
| Cross bred ewes | **🞎** |  | Medium (20.6-22.5 microns) | **🞎** |
|  |  |  | Strong (≥22.6 microns) | **🞎** |

| Cropping 🞎 | Area cropped (ha) |  | |  |
| --- | --- | --- | --- | --- |
|  | Crop types: | Summer: |  | |
|  |  | Winer: |  | |
| Other (Please specify) |  |  |  | |

**Flock details**

**Stock numbers on (date) ___________________**

| Merino | **🞎** | Total number of sheep |  |
| --- | --- | --- | --- |
| Cross bred | **🞎** | Number of ewes |  |
| Other | **🞎** | Number of wethers |  |
| (Please specify |  | Number of lambs |  |
|  |  | Number of rams |  |
| Flock bloodline |  |  |  |

**SECTION 2 - Flock details and management:**

**Sheep purchased/introductions**

Is the flock self-replacing 🞎 Yes 🞎 No

Have you regularly introduced **rams** over the past **5 years** 🞎 Yes 🞎 No

| If yes, how many |  |
| --- | --- |
| From how many sources |  |

| **Frequency of flock and hoof inspections** | Never | Weekly | Monthly | Every 3  months | Every 6  months | Other (Please specify) |
| --- | --- | --- | --- | --- | --- | --- |
| How frequently do you observe the flock for lameness | 🞎 | 🞎 | 🞎 | 🞎 | 🞎 |  |
|  |  |  |  |  |  |  |
| How frequently do you conduct hoof inspections | 🞎 | 🞎 | 🞎 | 🞎 | 🞎 |  |
| What percentage of the flock has poor hoof conformation? | | | |  | |  |

Which (if any) of the following conditions have been detected in the flock in the past year

Footrot – virulent 🞎

Footrot – benign 🞎

Hoof abscesses 🞎

Shelly hoof 🞎

Scald 🞎

What is the frequency of detection?

**SECTION 3 - Footrot history**

| If footrot has been diagnosed, what year was the diagnosis |  |
| --- | --- |
| Which year did you suspect the flock became infected? |  |
| What has been the duration of footrot infection on the property? |  |

What was the HIGHEST PERCENTAGE (%) of animals affected by footrot in the flock for each of the past 3 years

| **Year** | **% of flock Affected** |
| --- | --- |
| **2015** |  |
| **2016** |  |
| **2017** |  |

**Percentage (%) of animals in the flock affected by disease in the past 12 months**

| **Season** | **Percent (%) of flock affected each season** | **Total number of animals in the flock** |
| --- | --- | --- |
| Spring |  |  |
| Summer |  |  |
| Autumn |  |  |
| Winter |  |  |

**Footrot treatment**

**Please tick (🗸) if you have used any of the following footrot treatments in the past 12 months and indicate the frequency used**

|  | ≤ Monthly | 6 monthly | Annually | Other  (Please specify) |
| --- | --- | --- | --- | --- |
| 🞎 Foot bathing | 🞎 | 🞎 | 🞎 |  |
| 🞎 Hoof trimming | 🞎 | 🞎 | 🞎 |  |
| 🞎 Topical antibiotic | 🞎 | 🞎 | 🞎 |  |
| 🞎 Parenteral antibiotic | 🞎 | 🞎 | 🞎 |  |
| 🞎 Vaccination | 🞎 | 🞎 | 🞎 |  |
| 🞎 Other (Please specify) | 🞎 | 🞎 | 🞎 |  |

| **Please tick (🗸) to indicate at what level of disease prevalence in the flock you would use a treatment for footrot (see above list for examples of footrot treatments).** | |
| --- | --- |
| Individual animals | 🞎 |
| ≤ 5 % flock prevalence | 🞎 |
| 5-10 % flock prevalence | 🞎 |
| ≥ 10 % flock prevalence | 🞎 |

Have you culled sheep due to footrot in the past 12 months 🞎 Yes 🞎 No

| If yes, how many |  |
| --- | --- |

Have you culled sheep due lameness or other hoof issues in the past 12 months 🞎 Yes 🞎 No

| If yes, how many |  |
| --- | --- |

**SECTION 4 – Biosecurity**

| Number of neighbouring properties |  |
| --- | --- |
| Number of neighbours with sheep |  |
| Number of neighbouring properties with footrot |  |

**Feral Animals**

| **Please tick (🗸) if present** | **Number seen in the past month** |
| --- | --- |
| 🞎 Goats |  |
| 🞎 Deer |  |

| **Please tick (🗸) if the following apply** | **Frequency over last 12 months** |
| --- | --- |
| 🞎 Straying of sheep onto the property |  |
| 🞎 Sharing rams with neighbours |  |
| 🞎 Shared infrastructure with neighbours (roads, yards etc.) |  |
| 🞎 Shared grazing with neighbouring sheep |  |
| 🞎 Shared grazing with other ungulates (goats, cattle etc.) |  |

| Do you inspect the feet of sheep prior to purchase | 🞎 Yes 🞎 No |
| --- | --- |
| Do you request an Animal Health Statement prior to purchase | 🞎 Yes 🞎 No |
| Do you quarantine new stock | 🞎 Yes 🞎 No |
| If yes, for how long |  |

Do you have a sheep proof fence around the property 🞎 Yes 🞎 No

**Additional comments/information:**

**SECTION 5 - Producer perception
Please select the box that reflects your opinion about the following statements regarding the benign and lesser virulent forms of footrot.**

| **General Statements** | **Strongly**  **Disagree** | **Disagree** | **Neither**  **Agree or disagree** | **Agree** | **Strongly**  **Agree** | **Don’t**  **Know** |
| --- | --- | --- | --- | --- | --- | --- |
| Quarantine procedures are important in preventing disease | 🞎 | 🞎 | 🞎 | 🞎 | 🞎 | 🞎 |
| Footrot is caused by a bacteria | 🞎 | 🞎 | 🞎 | 🞎 | 🞎 | 🞎 |
| It is important to examine the feet of sheep prior to purchase | 🞎 | 🞎 | 🞎 | 🞎 | 🞎 | 🞎 |
| I am confident in my ability to identify sheep with footrot | 🞎 | 🞎 | 🞎 | 🞎 | 🞎 | 🞎 |

**Please select the box that reflects your opinion about the following statements regarding the benign and lesser virulent forms of footrot.**

| **Comment** | **Strongly**  **disagree** | **Disagree** | **Neither**  **agree or disagree** | **Agree** | **Strongly**  **agree** | **Don’t know** |
| --- | --- | --- | --- | --- | --- | --- |
| I consider hoof health an important animal health issue in the NSW sheep industry | 🞎 | 🞎 | 🞎 | 🞎 | 🞎 | 🞎 |
| I consider footrot a MINOR problem on the property | 🞎 | 🞎 | 🞎 | 🞎 | 🞎 | 🞎 |
| I consider footrot a MAJOR problem on the property | 🞎 | 🞎 | 🞎 | 🞎 | 🞎 | 🞎 |
| Poor hoof health has a negative impact on the welfare of affected sheep | 🞎 | 🞎 | 🞎 | 🞎 | 🞎 | 🞎 |
| Footrot is difficult to eradicate | 🞎 | 🞎 | 🞎 | 🞎 | 🞎 | 🞎 |
| Sheep on my property are at risk of footrot from infected neighbouring animals | 🞎 | 🞎 | 🞎 | 🞎 | 🞎 | 🞎 |
| lameness causes production losses (i.e. reduced wool and carcase weight) | 🞎 | 🞎 | 🞎 | 🞎 | 🞎 | 🞎 |
| I consider footrot a significant source of economic loss to the NSW sheep industry | 🞎 | 🞎 | 🞎 | 🞎 | 🞎 | 🞎 |
| There is sufficient online government resources about footrot | 🞎 | 🞎 | 🞎 | 🞎 | 🞎 | 🞎 |
| I am happy with the services and information offered by GOVERNMENT veterinarians | 🞎 | 🞎 | 🞎 | 🞎 | 🞎 | 🞎 |
| I am happy with the services and information offered by PRIVATE veterinarians | 🞎 | 🞎 | 🞎 | 🞎 | 🞎 | 🞎 |
| There is a need for additional government veterinary resources to manage footrot | 🞎 | 🞎 | 🞎 | 🞎 | 🞎 | 🞎 |
| Current methods of footrot management are cost effective | 🞎 | 🞎 | 🞎 | 🞎 | 🞎 | 🞎 |
| Current methods of footrot management are time effective | 🞎 | 🞎 | 🞎 | 🞎 | 🞎 | 🞎 |

**Supplementary Table 2.** Matrix with the Spearman’s rank correlation coefficients for the explanatory variables. Explanatory variables were deemed colinear if they yielded a value ˃0.7.

| **Explanatory variable** | **Explanatory variable** | | | | | | | | | |
| --- | --- | --- | --- | --- | --- | --- | --- | --- | --- | --- |
|  | Farm size | Average rainfall | Topography | Merino sheep present | Total no. sheep | Self-replacing flock | Ram sources | Lameness inspection | Poor hoof conformation | Treated when |
| Farm size | 1.00 | -0.11 | 0.02 | 0.20 | 0.48 | 0.30 | <-0.01 | -0.03 | 0.28 | 0.08 |
| Average rainfall | -0.11 | 1.00 | 0.60 | 0.09 | 0.12 | -0.13 | -0.19 | 0.09 | 0.33 | -0.01 |
| Topography | 0.02 | 0.60 | 1.00 | -0.06 | 0.38 | -0.16 | -0.01 | 0.12 | 0.35 | 0.35 |
| Merino sheep | 0.20 | 0.09 | -0.06 | 1.00 | 0.33 | 0.34 | 0.00 | 0.15 | 0.00 | -0.13 |
| Total no. sheep | 0.48 | 0.12 | 0.38 | 0.33 | 1.00 | 0.08 | 0.23 | 0.15 | 0.23 | 0.25 |
| Self-replacing flock | 0.30 | -0.13 | -0.16 | 0.34 | 0.08 | 1.00 | 0.26 | -0.12 | -0.26 | 0.14 |
| Ram sources | <-0.01 | -0.19 | -0.01 | 0.00 | 0.23 | 0.26 | 1.00 | 0.29 | -0.16 | <-0.01 |
| Lameness inspection | -0.03 | 0.09 | 0.12 | 0.15 | 0.15 | -0.12 | 0.29 | 1.00 | 0.00 | 0.35 |
| Poor hoof conformation | 0.28 | 0.33 | 0.38 | 0.00 | 0.23 | -0.26 | -0.16 | 0.00 | 1.00 | -0.01 |
| Treated when | 0.08 | -0.01 | 0.35 | -0.13 | 0.25 | 0.14 | <-0.01 | 0.35 | -0.01 | 1.00 |

**Supplementary Table 1.** Continued

| **Explanatory variable** | **Explanatory variable** | | | | | | | | | |
| --- | --- | --- | --- | --- | --- | --- | --- | --- | --- | --- |
|  | Farm size | Average rainfall | Topography | Merino sheep present | Total no. sheep | Self-replacing flock | Ram sources | Lameness inspection | Poor hoof conformation | Treated when |
| Neighbour with footrot | 0.15 | -0.27 | 0.06 | 0.00 | 0.36 | -0.03 | 0.08 | 0.28 | -0.13 | 0.35 |
| Feral animals | 0.15 | 0.09 | 0.28 | 0.16 | 0.00 | -0.07 | -0.03 | 0.05 | 0.00 | 0.08 |
| Straying sheep | 0.14 | 0.21 | 0.38 | 0.00 | 0.38 | 0.08 | 0.11 | 0.31 | 0.23 | 0.63 |
| Inspect feet at purchase | -0.16 | 0.11 | -0.02 | 0.09 | -0.09 | -0.07 | <-0.01 | -0.1 | 0.09 | -0.46 |
| Request AHS | -0.17 | 0.13 | <-0.01 | -0.40 | -0.26 | 0.02 | <-0.01 | 0.05 | -0.08 | 0.02 |
| Quarantine new sheep | 0.01 | 0.14 | -0.02 | 0.30 | 0.29 | 0.14 | -0.15 | 0.01 | -0.09 | -0.19 |
| Sheep proof fence | -0.01 | -0.02 | -0.34 | -0.02 | -0.43 | <-0.01 | 0.01 | 0.05 | -0.26 | -0.08 |

**Supplementary Table 2:** The values from the Hosmer-Lemeshow test for the outcome variables of the presence of footrot and the presence of other hoof diseases.

| **Outcome variable** | **Statistic** | **Degrees of freedom** | **P-value** |
| --- | --- | --- | --- |
| Presence of footrot | 0.89 | 2 | 0.64 |
|  |  |  |  |
| Presence of other hoof diseases | 0.35 | 2 | 0.84 |
